# All Expanded Criteria Donor Kidneys are Equal But are Some More Equal Than Others? A Population-Cohort Analysis of UK Transplant Registry Data

**DOI:** 10.3389/ti.2023.11421

**Published:** 2023-09-04

**Authors:** Kamlesh Patel, Anna Brotherton, Daoud Chaudhry, Felicity Evison, Thomas Nieto, Dilan Dabare, Adnan Sharif

**Affiliations:** ^1^ Department of Nephrology and Transplantation, University Hospitals Birmingham, Birmingham, United Kingdom; ^2^ School of Medical and Dental Sciences, University of Birmingham, Birmingham, United Kingdom; ^3^ Data Science Team, Research Development and Innovation, University Hospitals Birmingham, Birmingham, United Kingdom; ^4^ Institute of Immunology and Immunotherapy, University of Birmingham, Birmingham, United Kingdom

**Keywords:** expanded criteria donors, deceased donation, kidney transplant outcomes, epidemiology, ECD

## Abstract

Survival outcomes for kidney transplant candidates based on expanded criteria donor (ECD) kidney type is unknown. A retrospective cohort study was undertaken of prospectively collected registry data of all waitlisted kidney failure patients receiving dialysis in the United Kingdom. All patients listed for their first kidney-alone transplant between 2000–2019 were included. Treatment types included; living donor; standard criteria donor (SCD); ECD^60^ (deceased donor aged ≥60 years); ECD^50–59^ (deceased donor aged 50–59 years with two from the following three; hypertension; raised creatinine and/or death from stroke) or remains on dialysis. The primary outcome was all-cause mortality, with time-to-death from listing analyzed using time-dependent non-proportional Cox regression models. The study cohort comprised 47,917 waitlisted kidney failure patients, of whom 34,558 (72.1%) received kidney transplantation. ECD kidneys (*n* = 7,356) were stratified as ECD^60^ (*n* = 7,009) or ECD^50–59^ (*n* = 347). Compared to SCD, both ECD^60^ (Hazard Ratio 1.126, 95% CI 1.093–1.161) and ECD^50–59^ (Hazard Ratio 1.228, 95% CI 1.113–1.356) kidney recipients have higher all-cause mortality. However, compared to dialysis, both ECD^60^ (Hazard Ratio 0.194, 95% CI 0.187–0.201) and ECD^50–59^ (Hazard Ratio 0.218, 95% CI 0.197–0.241) kidney recipients have lower all-cause mortality. ECD kidneys, regardless of definition, provide equivalent and superior survival benefits in comparison to remaining waitlisted.

## Graphical Abstract

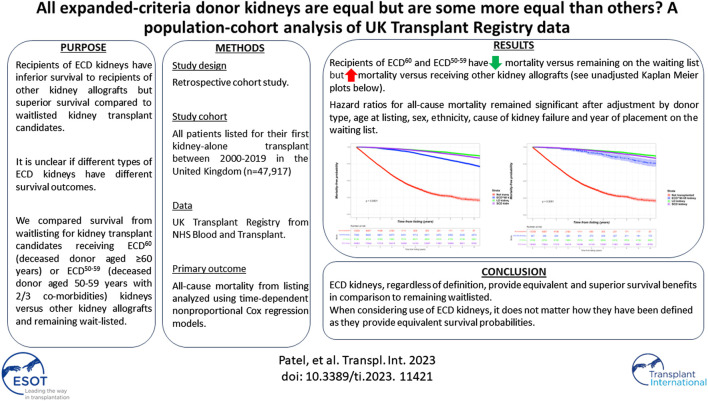

## Introduction

A broadening pool of donor kidneys are being utilized to bridge the gap between supply versus demand to facilitate more kidney transplantation. This includes expanded criteria donor (ECD) kidneys, which are defined based upon one of the following two conditions; either the deceased donor is aged ≥60 years or the deceased donor is aged between 50 and 59 years and fulfils any two of the following three criteria: 1) cause of death is cerebrovascular accident; 2) preexisting history of systemic hypertension; and 3) terminal serum creatinine >1.5 mg/dL (hereby referred to as ECD^60^ or ECD^50–59^, respectively) [1]. Defined by historical data from the United States, ECD kidneys are associated with increased risk of graft failure compared with standard criteria donor (SCD) kidneys by 70% (relative hazard ratio 1.70) [2]. Although kidney donor profile index (KDPI) now provides transplant professionals with additional information, this basic stratification of SCD versus ECD kidney has been adopted in other countries including the United Kingdom in allocation of kidneys and counselling of patients.

Although studies confirm lower survival rates versus other kidney allografts, recipients of ECD kidneys have improved survival compared with waitlisted dialysis-treated patients. In a systematic review and meta-analysis of 48 published cohort studies, compared to remaining on dialysis any type of kidney allograft was superior from an all-cause mortality perspective and this included ECD kidney transplantation versus remaining waitlisted [3]. However, at present any potential kidney transplant candidate is counselled generically about the outcomes associated with ECD kidneys versus alternative options, with no differentiation made between different ECD kidney types. This is due to a lack of any comparative data comparing any patient and/or graft survival difference between the two ECD classifications. With ECD kidneys increasing as a proportion of all deceased donor kidneys, clarifying any survival difference between different types of ECD kidneys is important.

Therefore, the aim of this study was to compare survival for waitlisted kidney transplant candidates receiving ECD^60^ versus ECD^50–59^ kidney transplantation in comparison to other forms of kidney allografts or remaining on the waiting list.

## Materials and Methods

### Study Cohort

A retrospective cohort study was undertaken of prospectively collected registry data related to all waitlisted kidney failure patients receiving dialysis in the United Kingdom. From 1 January 2000 until 30 September 2019 inclusive, all patients who were either listed and received their primary kidney-alone transplant versus those who were listed but never received a kidney transplant were included in the study. No formal sample size estimate was conducted as all eligible patient records were used. 31 December 2020 was considered the study end. The study is reported as per STROBE guidance [4].

### Study Variables

The following study variables were available for all patients; age (at listing and at transplantation), sex, ethnicity [classified as white, black, Asian (Indo-Asian), other, known], primary cause of kidney failure (classified as diabetes, glomerulonephritis, hypertension, other separate, polycystic kidney disease, pyelonephritis/reflux nephropathy, unknown/missing), year of listing, and waiting time.

Donor kidneys were stratified into living donors or any deceased donor (inclusive of donors after brain or circulatory death) further stratified into standard criteria donors (SCD), expanded criteria donor from deceased donors aged ≥60 years without comorbidities (ECD^60^) or expanded criteria donor from deceased donors aged between 50 and 59 years with two comorbidities among hypertension, death from cerebrovascular accident, or terminal serum creatinine levels >1.5 mg/dL (ECD^50–59^). The remaining waitlisted kidney transplant candidates did not proceed for transplantation and remained on dialysis.

### Outcomes

The primary outcome of interest was all-cause mortality. The survival analysis was conducted according to the intention-to-treat principle; therefore, patients were not dropped from the analysis if they were removed from the waiting list or if transplantation subsequently failed. Secondary outcomes included death-censored graft loss.

### Statistical Analysis

For baseline demographics, continuous variables were reported as medians and interquartile ranges (IQRs) and compared between groups using Mann-Whitney tests. Ordinal factors were also compared using Mann-Whitney tests, whilst nominal factors were analysed using Fisher’s exact tests or Chi-square tests for those with two or more than two categories, respectively. Missing data underwent list-wise deletion.

Survival was analysed as time from initial placement on the waiting list to death, with data censored at loss of follow up or on 31 December 2020. Unadjusted survival-free probability was analysed by generation of Kaplan–Meier curves. After testing for violations of the proportional hazard assumption, time-to-death was modelled using non-proportional hazard Cox regression models with transplantation handled as a time-dependent covariate. Using this approach, all patients contribute data for time at risk (and death if it occurs) to the non-transplant group starting at study entry with those receiving a transplant switching time at risk (and death if it occurs) to the transplant group starting at the time of surgery (this forms the time-dependent transplant covariate in the model). Mortality hazard ratios were computed for the transplant recipients compared with those on the waiting list. We explored adjusted models factoring for age at listing, sex, ethnicity, cause of kidney failure and year of placement on the waiting list. An extended non-proportional hazard Cox regression model with both transplantation and graft loss handled as time-dependent variables was also included. Time to graft loss models were conducted using weighted Cox regression models and adjusted for age at listing, sex, ethnicity, cause of kidney failure, waiting time, year of placement of the waiting list, level of HLA mismatches, delayed graft function and 1-year rejection.

All analyses were done using R 4.0.4 (R Foundation for Statistical Computing, Vienna, Austria).

### Approvals

National Health Service Blood and Transplant (NHSBT) obtains informed consent from all patients undergoing solid organ transplantation in the United Kingdom for data collection and subsequent analyses. Study proposals are reviewed and approved by the kidney advisory group on behalf of NHSBT as IRB approval (ref: HD29035) before data dissemination.

## Results

### Study Cohort

**FIGURE 1:**
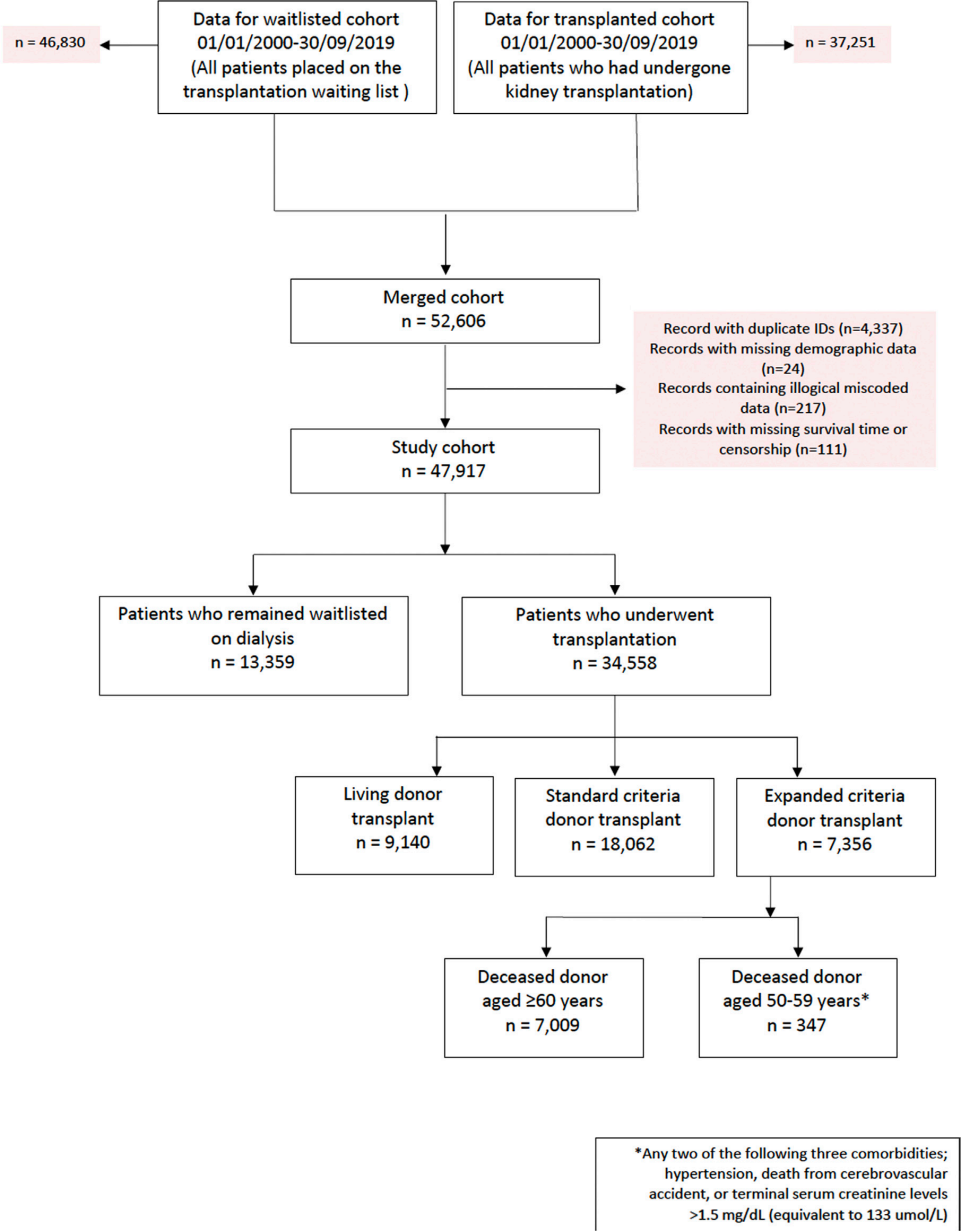
PRISMA flowchart of study cohort.

The original cohort obtained from NHSBT contained records from two datasets between 1 January, 2000 until 30 September, 2019; kidney failure patients listed who received a kidney transplant (*n* = 37,251) and kidney failure patients listed for transplantation (*n* = 46,830). After combining both datasets, duplicated records and/or cases with missing demographic data were excluded. This left 47,917 kidney failure patients to form our study cohort, of whom 34,558 (72.1%) subsequently received their first kidney transplant after waitlisting (living donors; *n* = 9,140, SCD; *n* = 18,062 and ECD; *n* = 7,356). From the deceased donor groups, 28.6% (*n* = 5,174) and 37.1% (*n* = 2,730) of SCD and ECD kidneys respectively were from donors after circulatory death. From the ECD recipient group, 7,009 were classified based upon donor aged ≥60 years (ECD^60^) while 347 were classified based upon donor aged between 50–59 years and additional criteria met (ECD^50–59^). This likely represents under ascertainment of ECD^50–59^ kidney allografts: while data completeness for donor cause of death or age were excellent at 100%, data completeness was only 67.3% for donor creatinine, for example. As this is an integral aspect of our analysis, to account for this limitation we have performed sub-group analyses after removal of all missing creatinine values to ensure the primary findings are replicated. See Figure 1 for the PRISMA flowchart of the study cohort.

Table 1 shows baseline demographics at the time of listing for the study cohort and identifies significant differences in baseline demographics between those that received different types of kidney allografts versus those that remained without transplantation. Most importantly, it confirms the significantly higher proportion of ECD kidneys allocated to older kidney transplant candidates. Table 2 compares waitlisted kidney transplant candidates who received ECD^50–59^ versus ECD^60^ kidneys. Kidney transplant candidates receiving ECD^50–59^ versus ECD^60^ kidneys were younger (both at waitlisting and surgery) and had different causes of kidney failure.

**TABLE 1:** Baseline demographics of study cohort.

Variable		LD kidney	SCD kidney	ECD kidney	Dialysis	*p*-value
N		9,140	18,062	7,356	13,359	—
Median Age at waitlisting in years (IQR)		43 (23)	45 (19)	57 (15)	53 (21)	<0.001
Percentage (*n*) patients aged ≥60 years at listing		13.9% (1,271)	13.6% (2,461)	41.4% (2,046)	33.7% (4,408)	<0.001
Percentage (*n*) patients aged ≥65 years at listing		6.6% (605)	6.2% (1,120)	22.5% (1,653)	20.3% (2,708)	<0.001
Percentage (*n*) patients aged ≥70 years at listing		1.8% (167)	1.7% (299)	7.9% (580)	8.0% (1,069)	<0.001
Sex	Male	61.4% (5,611)	62.7% (11,326)	64.2% (4,719)	61.0% (8,143)	<0.001
	Female	38.6% (3,529)	37.3% (6,736)	35.8% (2,637)	39.0% (5,216)	
Ethnicity	White	82.6% (7,550)	75.3% (13,593)	75.2% (5,532)	71.6% (9,564)	<0.001
	Asian	8.8% (808)	13.4% (2,418)	13.5% (990)	15.5% (2,072)	
	Black	4.8% (436)	7.7% (1,383)	7.5% (554)	9.0% (1,198)	
	Other	2.8% (252)	2.7% (496)	3.0% (219)	3.1% (416)	
	Unknown	1.0% (94)	1.0% (172)	0.8% (61)	0.8% (109)	
Cause of kidney failure	Diabetes	7.2% (659)	7.5% (1,351)	12.3% (903)	27.6% (3,681)	<0.001
	Glomerulonephritis	6.6% (602)	6.8% (1,231)	6.3% (462)	3.8% (511)	
	Hypertension	4.7% (431)	5.3% (950)	6.7% (491)	4.7% (633)	
	Other Separate	31.8% (2,905)	27.2% (4,911)	24.7% (1,815)	20.9% (2,787)	
	Polycystic Kidney	8.9% (810)	11.5% (2,072)	12.4% (909)	6.3% (845)	
	Pyelonephritis/reflux	6.9% (629)	7.8% (1,411)	5.9% (431)	4.4% (592)	
	Unknown/Missing	34.0% (3,104)	34.0% (6,136)	31.9% (2,345)	32.3% (4,310)	

LD, living donor; SCD, standard criteria donor; ECD, expanded criteria donor; IQR, interquartile range.

**TABLE 2:** Characteristics of recipient receiving ECD kidneys.

Variable		All ECD kidney	ECD^50–59^	ECD^60^	*p*-value
Percentage (*n*)		100% (7,356)	4.7% (347)	95.3% (7,009)	—
Median Age at waitlisting in years (IQR)		57 (15)	49 (17)	58 (15)	<0.001
Median Age at transplantation in years (IQR)		60 (14)	53 (18)	60 (14)	<0.001
Sex	Male	64.2% (4,719)	65.4% (227)	64.1% (4,492)	0.614
	Female	35.8% (2,637)	34.6% (120)	35.9% (2,517)	
Ethnicity	White	75.2% (5,532)	71.% (247)	75.4% (5,285)	0.093
	Asian	13.5% (990)	14.4% (50)	13.4% (940)	
	Black	7.5% (554)	10.7% (37)	7.4% (517)	
	Other	3.0% (219)	2.3% 8)	3.0% (211)	
	Unknown	0.8% (61)	1.4% 5)	0.8% (56)	
Cause of kidney failure	Diabetes	12.3% (903)	7.5% (26)	12.5% (877)	0.008
	Glomerulonephritis	6.3% (462)	9.8% (34)	6.1% (428)	
	Hypertension	6.7% (491)	6.1% (21)	6.7% (470)	
	Other Separate	24.7% (1,815)	23.1% (80)	24.8% (1,735)	
	Polycystic Kidney	12.4% (909)	13.5% (47)	12.3% (862)	
	Pyelonephritis/reflux	5.9% (431)	7.8% (47)	5.8% (404)	
	Unknown/Missing	31.9% (2,345)	32.3% (112)	31.9% (2,233)	
Waiting time in days (IQR)		896 (988)	844 (1,128)	899 (978)	0.949

ECD, expanded criteria donor; IQR, interquartile range.

### Mortality Events

**FIGURE 2:**
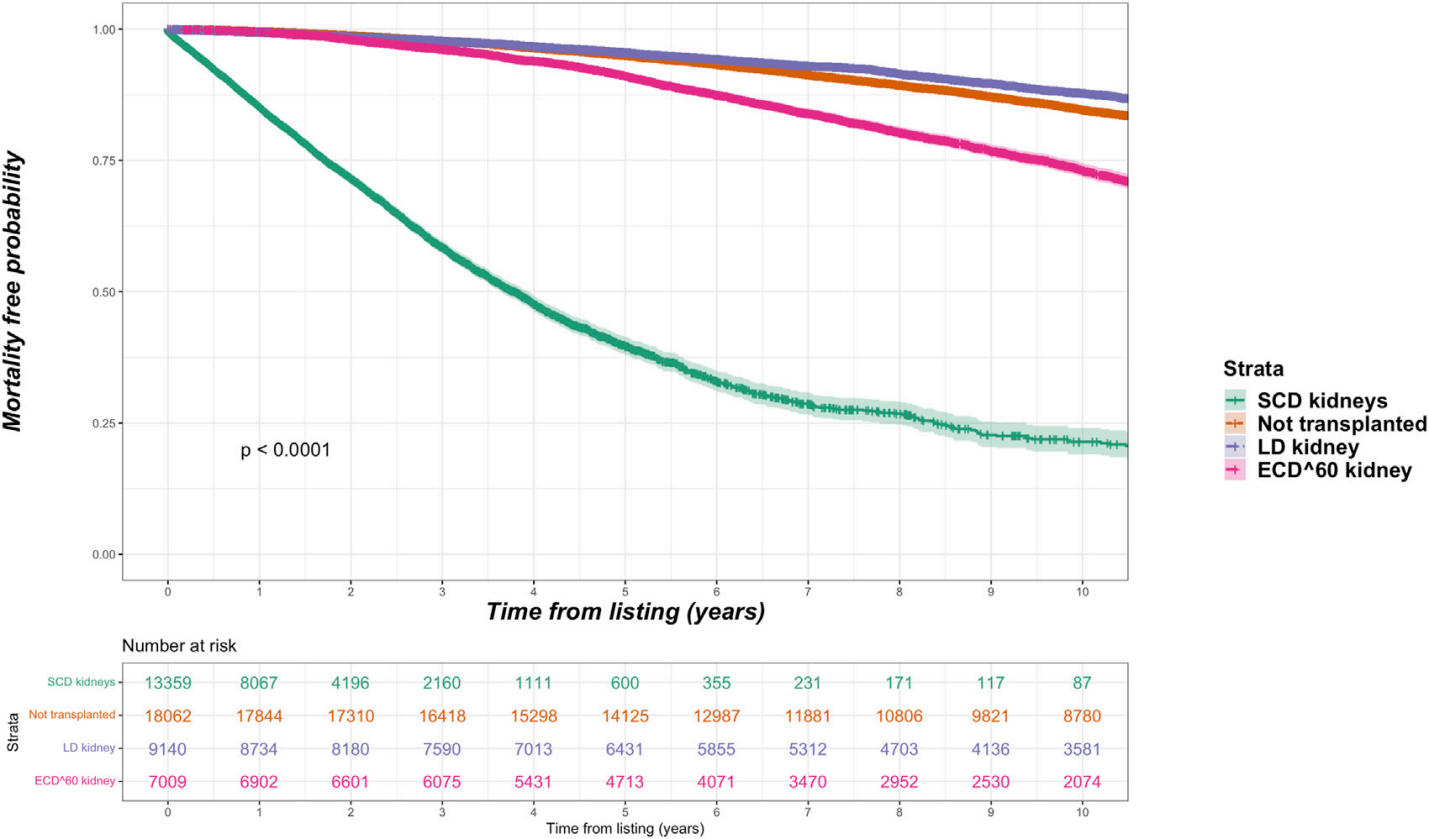
Unadjusted Kaplan-Meir plot of mortality free survival comparing recipients of ECD^60^ kidneys versus all other kidney allografts versus remaining waitlisted on dialysis.

**FIGURE 3:**
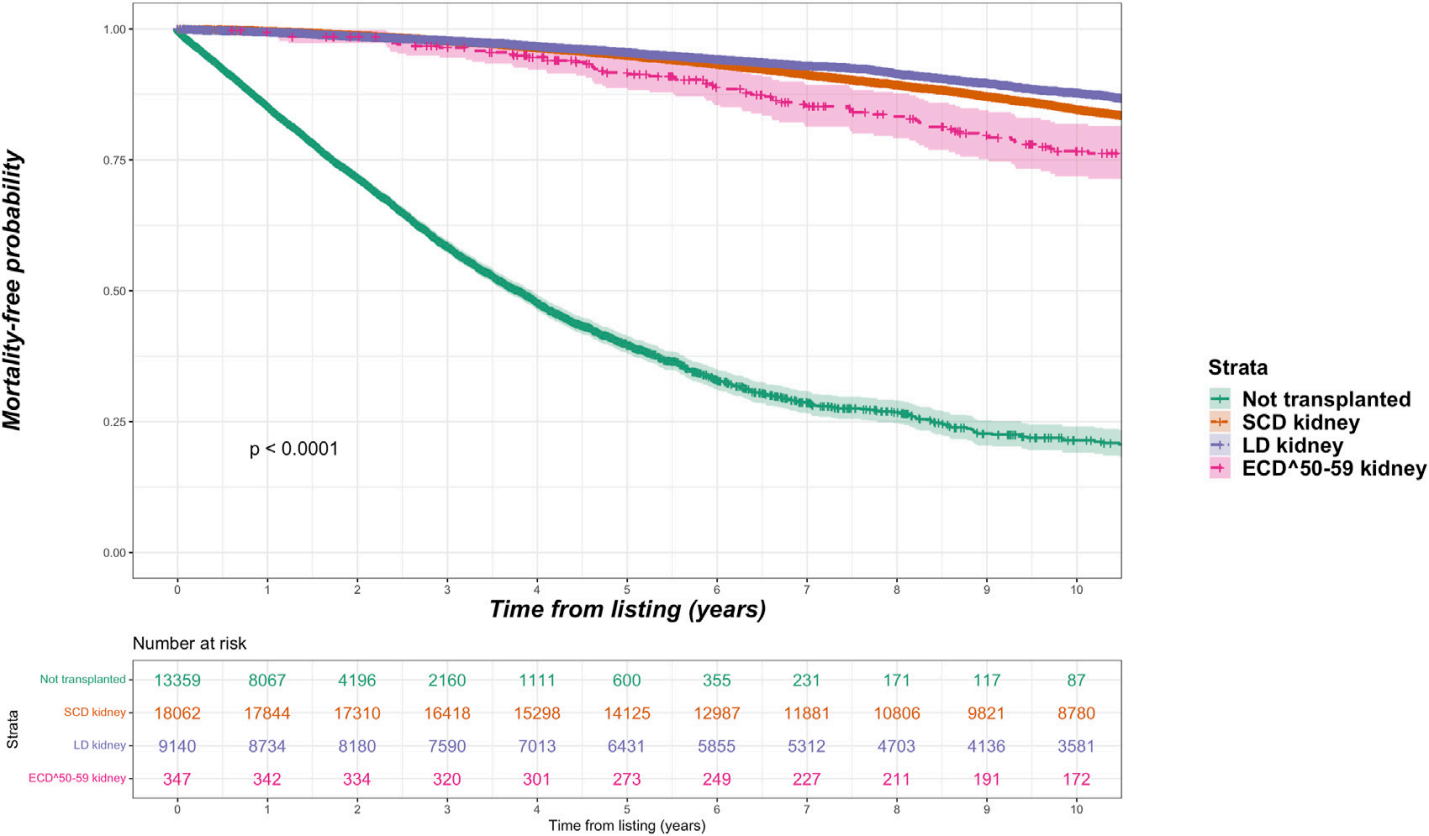
Unadjusted Kaplan-Meir plot of mortality free survival comparing recipients of ECD^50–59^ kidneys versus all other kidney allografts versus remaining waitlisted on dialysis.

Overall, the kidney failure group that was listed but did not receive kidney transplantation had 4,003 deaths (42.8% of cohort) versus 6,695 deaths (24.0% of cohort) among the listed group that received kidney transplantation. For the transplant group, 1,127 deaths occurred after living donor transplantation (14.1% of living donor cohort), 3,701 deaths after SCD transplantation (25.8% of SCD cohort) and 1,867 deaths after ECD transplantation (34.0% of ECD cohort). Among the ECD cohort, 103 deaths were in the ECD^50–59^ cohort (4.7% of all deaths after ECD kidney transplantation) and 1,764 in the ECD^60^ cohort. The total period of follow up for the entire cohort was 349,964 patient-years, with median follow up after waitlisting of 5.8 years. Unadjusted Kaplan-Meir plots for mortality stratified by ECD^60^ or ECD^50–59^ kidneys versus other treatment options are shown in Figures 2, 3, respectively.

### Unadjusted and Adjusted Graft Survival (Death-Censored) Using Weighted Cox Regression

**FIGURE 4:**
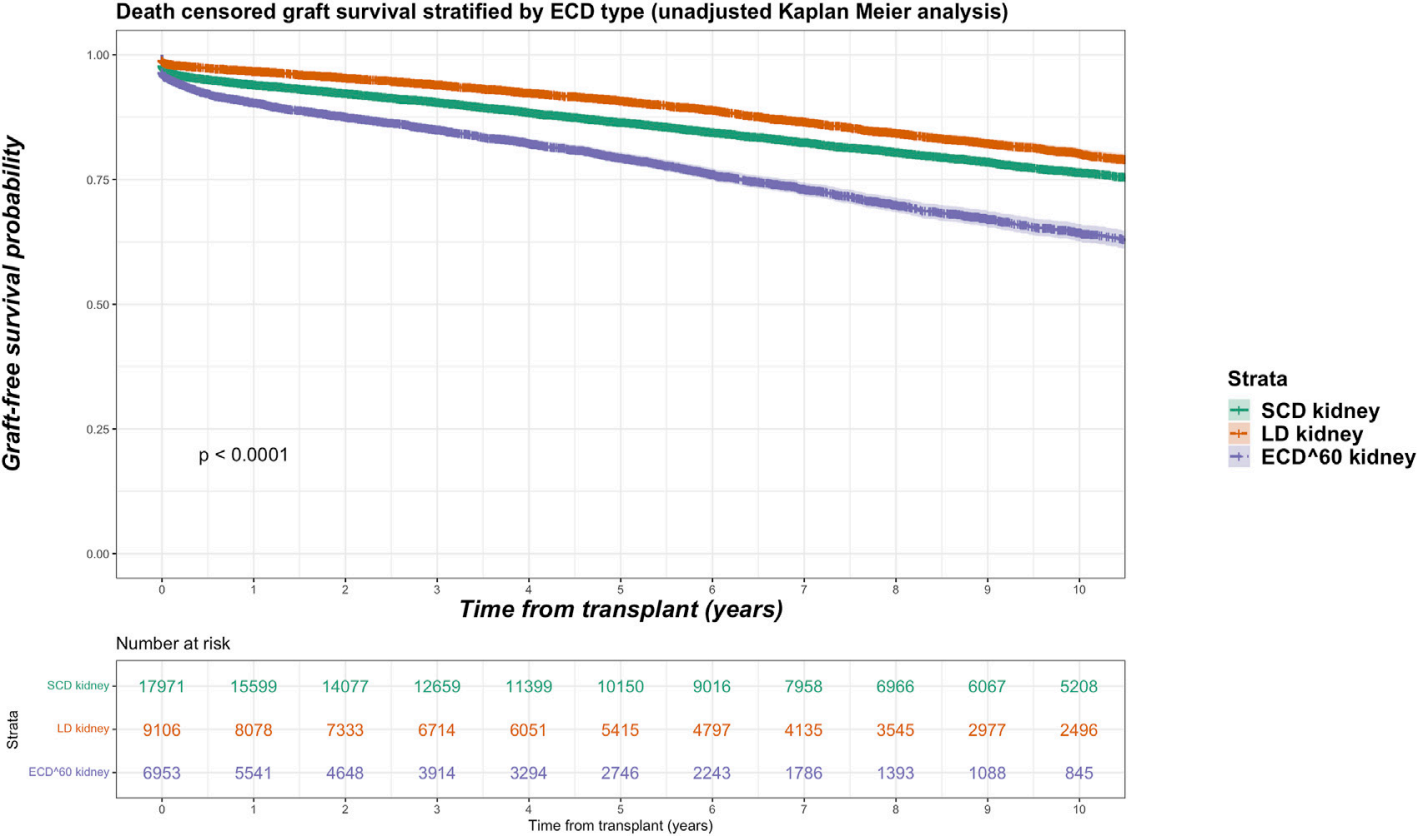
Unadjusted Kaplan-Meir plot of graft loss free survival comparing recipients of ECD^60^ kidneys versus all other kidney allografts.

**FIGURE 5:**
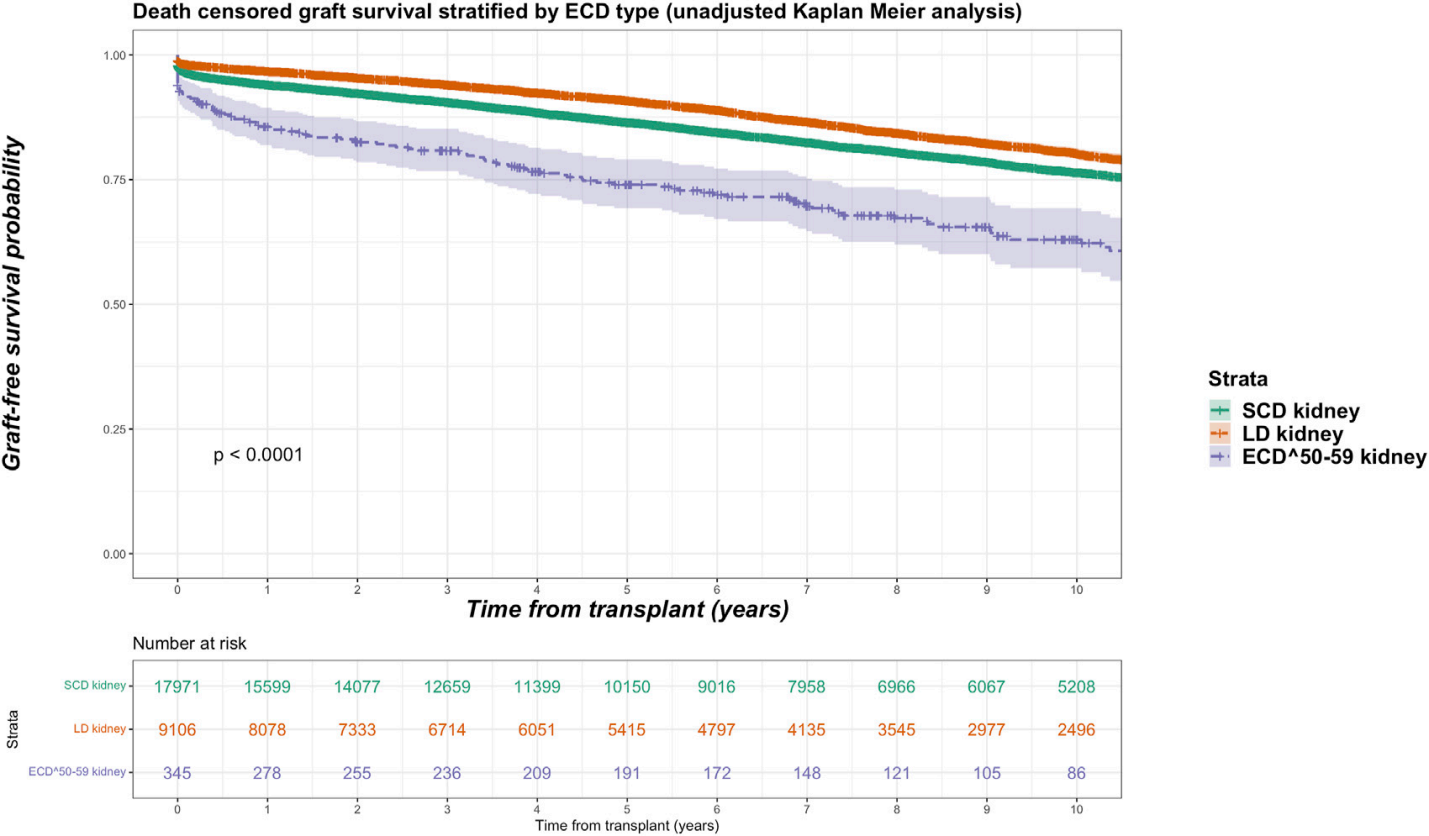
Unadjusted Kaplan-Meir plot of graft loss free survival comparing recipients of ECD^50–59^ kidneys versus all other kidney allografts.

**FIGURE 6:**
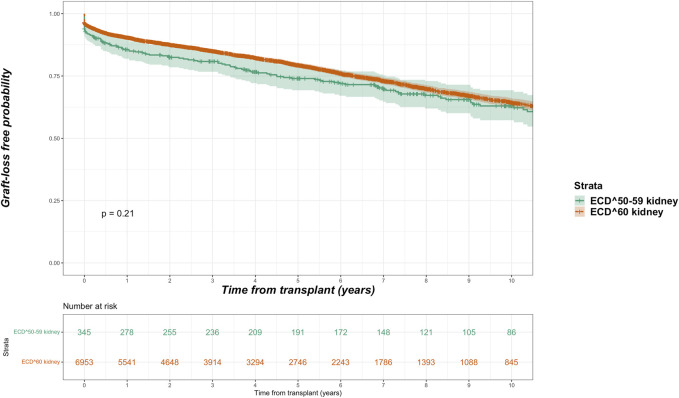
Unadjusted Kaplan-Meir plot of graft loss free survival comparing recipients of ECD^60^ versus ECD^50–59^ kidneys.

Among the kidney transplant recipients (*n* = 34,375), there were a total of 6,893 (20.1%) death-censored graft losses over the follow up period. Graft losses stratified by donor type were living donor (*n* = 1,440, 15.8%), SCD (*n* = 3,658, 20.4%) and ECD (*n* = 1,795, 24.6%). Splitting ECD into the different classifications, graft losses occurred in 24.0% (*n* = 1,670) of ECD^60^ kidneys versus 36.2% (*n* = 125) of ECD^50–59^ kidneys. Unadjusted Kaplan-Meir plots for death-censored graft loss stratified by ECD^60^ or ECD^50–59^ kidneys versus other transplant treatment options are shown in Figures 4, 5, respectively, with a comparison between the two ECD types shown in Figure 6.

In adjusted models, compared to receiving a SCD kidney, receiving any ECD kidney was associated with an increased risk for graft loss (HR 2.580, 95% CI 2.153–3.092, *p* < 0.001). After splitting ECD kidneys into the different classifications, compared to SCD kidneys both ECD^60^ kidneys (HR 2.638, 95% CI 2.202–3.161 *p* < 0.001) and ECD^50–59^ kidneys (HR 1.836, 95% CI 1.179–2.859 *p* = 0.007) were associated with increased risk for graft loss. When compared to each other, ECD^60^ kidneys had equivalent risk for graft loss against ECD^50–59^ kidneys (HR 0.905, 95% CI 0.597–1.373, *p* = 0.640).

### Non-Proportional Hazards Cox Regression Model With Transplantation a Time-Dependent Covariate

In a non-proportional hazard Cox regression model using a time-dependent analysis, with transplantation handled as a time-dependent covariate, recipients of ECD^60^ kidneys had increased all-cause mortality compared to SCD kidneys (HR 1.126, 95% CI 1.093–1.161, *p* < 0.001) but lower all-cause mortality versus remaining on the waiting list (HR 0.194, 95% CI 0.187–0.201, *p* < 0.001) as per Table 3. Recipients of ECD^50–59^ kidneys also had increased all-cause mortality compared to SCD kidneys (HR 1.228, 95% CI 1.113–1.356, *p* < 0.001) but lower all-cause mortality compared to remaining on the waiting list (HR 0.218, 95% CI 0.197–0.241, *p* < 0.001).

**TABLE 3:** Non-proportional hazard Cox model of predictors for mortality after kidney transplantation with either dialysis or SCD as reference (fully adjusted model with transplantation handled as a time varying covariate).

Variable			HR (95% CI)	Variable		HR (95% CI)
ECD^50–59^ kidneys	Treatment	Dialysis	1.000	Treatment	SCD	1.000
		ECD^50–59^	**0.218 (0.197–0.241)**		ECD^50–59^	**1.228 (1.113–1.356)**
		SCD	**0.177 (0.171–0.183)**		Dialysis	**5.644 (5.452–5.843)**
		LD	**0.145 (0.139–0.151)**		LD	**0.818 (0.790–0.848)**
ECD^60^ kidneys	Treatment	Dialysis	1.000	Treatment	SCD	1.000
		ECD^60^	**0.194 (0.187–0.201)**		ECD^60^	**1.126 (1.093–1.161)**
		SCD	**0.172 (0.166–0.178)**		Dialysis	**5.809 (5.615–6.008)**
		LD	**0.142 (0.137–0.149)**		LD	**0.827 (0.799–0.856)**

LD, living donor; SCD, standard criteria donor; ECD, expanded criteria donor; HR, hazard ratio; CI, confidence interval. Analysis adjusted by donor type, age at listing, sex, ethnicity, cause of kidney failure and year of placement on the waiting list.

Bold values indicate statistical significance.

### Non-Proportional Hazards Cox Regression Model With Both Transplantation and Graft Loss Time-Dependent Covariate

We conducted a non-proportional Cox regression analysis with both transplantation and graft loss factored as time-dependent covariates. In this extended model, compared to SCD kidney transplantation, both ECD^60^ (HR 1.102, 95% CI 1.084–1.120, *p* < 0.001) and ECD^50–59^ (HR 1.201, 95% CI 1.139–1.266, *p* < 0.001) kidney recipients had increased risk for all-cause mortality, but lower all-cause mortality compared to remaining on dialysis (ECD^60^: HR 0.198, 95% CI 0.192–0.204, *p* < 0.001 and ECD^50–59^: HR 0.221, 95% CI 0.208–0.241, *p* < 0.001).

### Sub-Analyses

In view of missing donor creatinine data, we undertook a sub-analysis excluding deceased donors with a missing donor creatinine to ensure no erroneous cross-over of ECD patients as SCD patients (see Supplementary Material). No difference was observed from our primary analysis and we found no evidence that the results were skewed by missing donor creatinine data.

## Discussion

Since its emergence, ECD kidneys have been a valuable source of allografts to bridge the gap between supply versus demand for waitlisted kidney transplant candidates to proceed with transplantation versus remaining on dialysis. While ECD kidney transplantation generally provides survival benefits versus remaining on dialysis, no data exists to ascertain any difference in survival dependent upon which type of ECD kidney is implanted. To the best of our knowledge, this is the first analysis to investigate this in a population-cohort analysis and demonstrates the following important observations; 1) both ECD^60^ and ECD^50–59^ kidneys demonstrate inferior patient and graft survival in comparison to SCD kidneys; 2) despite the inferior survival comparison to SCD kidneys, recipients of both ECD^60^ or ECD^50–59^ kidneys have significantly lower all-cause mortality versus being waitlisted and never being transplanted, and; 3) there is no survival difference when comparing both ECD kidney allografts to each other.

The literature provides conflicting data with regards to survival benefits afforded by receiving ECD kidneys, especially among older kidney transplant candidates. The latter is important as our data confirms ECD kidney allocation is prioritized for older kidney transplant candidates to be the preferred recipient. A previous systematic review of published studies suggested ECD kidneys should be allocated for older (aged ≥40 years) kidney transplant candidates or those receiving their first allograft [5]. Prioritizing ECD kidneys for older recipients, by ignoring immunology-based allocation, has been a successful strategy implemented by the Eurotransplant Senior program and demonstrates favourable 5-year outcomes [6]. Our data are broadly consistent with these observations, showing survival benefit for ECD kidney transplantation versus remaining waitlisted independent of age. However, more recent study findings challenge this widely accepted opinion. Hellemans et al. [7] studied a Belgian cohort of 3,808 waitlisted kidney transplant candidates, of whom 3,382 subsequently received a deceased donor kidney transplant. Older recipients (aged ≥65 years) of ECD kidney transplants did not have a survival benefit when compared to remaining on dialysis in contrast to older recipients of SCD kidney transplants. All kidney transplant candidates had increased mortality risk post-operatively with subsequent survival benefit except for older recipients who received an ECD kidney transplant.

The outcomes from Hellemans et al. are surprising as previous studies suggest favorable all-cause mortality benefits from receiving ECD kidneys in European countries versus the United States. Querard et al. [8] conducted a systematic review and meta-analysis of 32 studies comparing survival outcomes between recipients of SCD versus ECD kidneys, with pooled 5-year patient survival probabilities 86.4% and 78.4%, respectively. A significant difference in mortality benefit was observed comparing European and North American studies, with 5-year pooled patient survival between SCD and ECD kidney recipients closer in European studies (90.3% and 85.3%, respectively) versus North American studies (83.6% and 73.4%, respectively). Despite this survival disparity, ECD kidney transplant outcomes remain favourable in the United States, where both Gill et al. [9] and Merion et al. [10] have observed prolonged time to survival benefits for recipients of ECD kidneys (especially among older and/or high-risk patients) but ultimate mortality advantage. Survival disparity may reflect differences in kidney failure survival, with high dialysis mortality observed in the United States skewing risk-versus-benefit ratios between the continents [11].

Considering the findings from Hellemans et al., our study is reassuring whilst providing new insights to the literature. This is important considering ECD kidneys now constitute over a third of deceased donor kidneys [12]. Our data confirms ECD kidneys, regardless of how they are defined, are a valuable source of deceased donor kidneys for waitlisted kidney transplant candidates. The survival difference between ECD^60^ and ECD^50–59^ kidneys are negligible, especially when compared to remaining on the waiting-list. Our data also shows any survival benefit is independent of age at listing, which is important as many national organ offering systems prioritize ECD kidneys for older kidney transplant candidates and our results support this strategy regardless of ECD type. Both ECD kidneys are associated with increased risk for death-censored graft loss, as seen in our results and from published reports [8]. However, many studies do not factor graft loss as a time-dependent covariate in their post-transplant models for mortality. Our results are encouraging as they confirm, even with increased risk of graft loss, patient survival benefit from receiving an ECD kidney is clear. Regardless of these benefits, optimizing use of ECD kidneys for selected recipients may be prudent. For example, ECD kidney allograft survival may be improved in the absence of circulating donor-specific antibody (*p* < 0.001) and cold ischemic times <12 h (*p* = 0.030) according to a French study [13]. Optimal utilization of ECD kidneys may also be stratified by recipient age, with studies suggesting recipients aged ≥60 years [14] or ≥65 years [7] be prioritized. However, 10-year population-average effects using propensity scores suggest minimal absolute effect of only 8 months (95% CI 2–14 months) quicker time to graft failure attributed to ECD kidneys [7]. Therefore, the absolute risk difference between SCD and ECD kidneys in the long-term may be marginal when compared to remaining on the waiting-list.

One question our study cannot answer is whether a kidney transplant candidate should decline any ECD kidney and wait for a “better” deceased donor offer (e.g., a SCD kidney). Data from the United States shows the benefit of accepting “marginal” kidneys based upon specific recipient characteristics [15]. We suggest the certainty of outcomes associated with receiving an ECD kidney transplant, weighed against the uncertainty of outcomes regarding when an appropriate repeat deceased donor offer will emerge, must be carefully considered by any kidney transplant candidate. This is important as declined kidney offers are not benign events. Husain et al., in a cohort study analyzing 280,041 wait-listed kidney transplant candidates in the United States, observed approximately 30% of candidates receiving at least one deceased donor offer declined on their behalf eventually died or were removed from the waiting-list before receiving a kidney allograft [16]. Whilst data from the United Kingdom is more reassuring, with post organ decline deaths or removal from the waiting list occurring in 4% and 12% of kidney transplant candidates after 1-year or 5-year, respectively [17], there is no guarantee that declining a kidney allograft in the hope for a “better” kidney will be successful or facilitate timely transplantation. We believe that despite the survival differences observed in our analyses between SCD and any ECD kidney, our data should provide reassurance to kidney transplant candidates offered ECD kidneys. This is because those being offered an ECD kidney do not have a choice between an ECD versus a SCD kidney; their choice is between kidney transplantation versus no kidney transplantation. We believe this is the fundamental choice that kidney transplant candidates must consider, especially older candidates who are primed through national organ allocation algorithms to be prioritized for ECD kidney offers. Considering the excess morbidity, mortality and costs related to dialysis therapies, limited financial resources from healthcare providers should focus on maximizing usage of all donated organs to avoid wastage of “marginal” organs which current evidence suggest provides a survival benefit to most (if not all) waitlisted kidney transplant candidates.

Our study benefits from being a contemporary analysis of a national population-cohort, compatible with the modern era of organ donation and kidney transplantation. The limitations of this study must be appreciated for accurate interpretation of the results. Missing donor-related data (e.g., terminal creatinine) means some deceased-donors may have been erroneously coded as SCD rather than ECD^50–59^ kidneys, leading to an under-estimate. This must be interpreted as a significant limitation of this analysis, with the potential to skew results erroneously as donor creatinine is one of the three classification criteria for an ECD kidney. Future studies must aim to minimise such missing data for robustness. While acknowledging this limitation, we have undertaken additional sub-analyses to provide some validation of our primary findings but this limitation regarding missing data must be appreciated when interpreting our results. As an intention-to-treat analysis, we did not factor for waitlisted kidney failure patients who were suspended or removed from the waiting list due to lack of fitness. Censoring patients at delisting would have yielded an overestimation of survival on dialysis as data from the United Kingdom confirms increased mortality associated for waitlisted kidney failure patients who experience any period of suspension [18]. This analysis comprised waitlisted kidney transplant candidates who either had their primary transplant or remained on dialysis; therefore it provides no targeted evidence in the setting of advanced chronic kidney disease or a failed kidney transplant exploring repeat transplantation. Lack of data relating to medical co-morbidities limits interpretation of survival probabilities in the setting of specific health burdens, which may tip the balance of more borderline risk versus benefit calculations for older candidates and ECD kidneys. This is a critically important limitation that should be overcome for future analyses. The binary use of ECD kidneys is a crude distinction. While still utilized, the use of Kidney Donor Profile Indexes in the United States since 2014 is common but may not be directly translatable to European cohorts [19]. Finally, this analysis has focused solely upon survival benefits associated with transplant surgery for kidney failure patients and overlooks the importance of quality of life which was beyond the scope of this study but is under investigation elsewhere [20].

To conclude, in this contemporary national cohort study of kidney failure patients listed for transplantation, proceeding with any type of ECD kidney transplant affords a survival benefit to kidney transplant candidates versus remaining on dialysis. Although associated with increased mortality compared to recipients of other kidney allografts, which is an important consideration for waitlisted candidates with realistic chances for a timely SCD or living donor transplant, ECD kidneys for the majority offers a valuable opportunity of kidney transplantation. While our data is reassuring, the caveat remains that survival benefits at a population-level must be translated to individual kidney transplant candidates with personalized risk counselling. Further analyses would be beneficial to provide more nuanced survival probability investigations in the context of medical co-morbidities. However, our data should provide reassurance to clinicians involved in the care of kidney failure patients that kidney transplantation using ECD kidneys provides an excellent opportunity to improve survival probabilities.

## Data Availability

Data for this analysis was acquired from the UK transplant registry, which is accessible upon reasonable request.

## Supplementary Material

Supplementray Figure S1 Unadjusted Kaplan-Meir plot of mortality free survival comparing recipients of ECD50-59 kidneys versus all other kidney allografts versus remaining waitlisted on dialysis (missing donor creatinines excluded).
