# Supplementary figures and images for: All Expanded Criteria Donor Kidneys are Equal But are Some More Equal Than Others? A Population-Cohort Analysis of UK Transplant Registry Data
Source: Transpl Int. 2023 Sep 4;36:11421. doi: 10.3389/ti.2023.11421 (PMC10505656; doi:10.3389/ti.2023.11421)

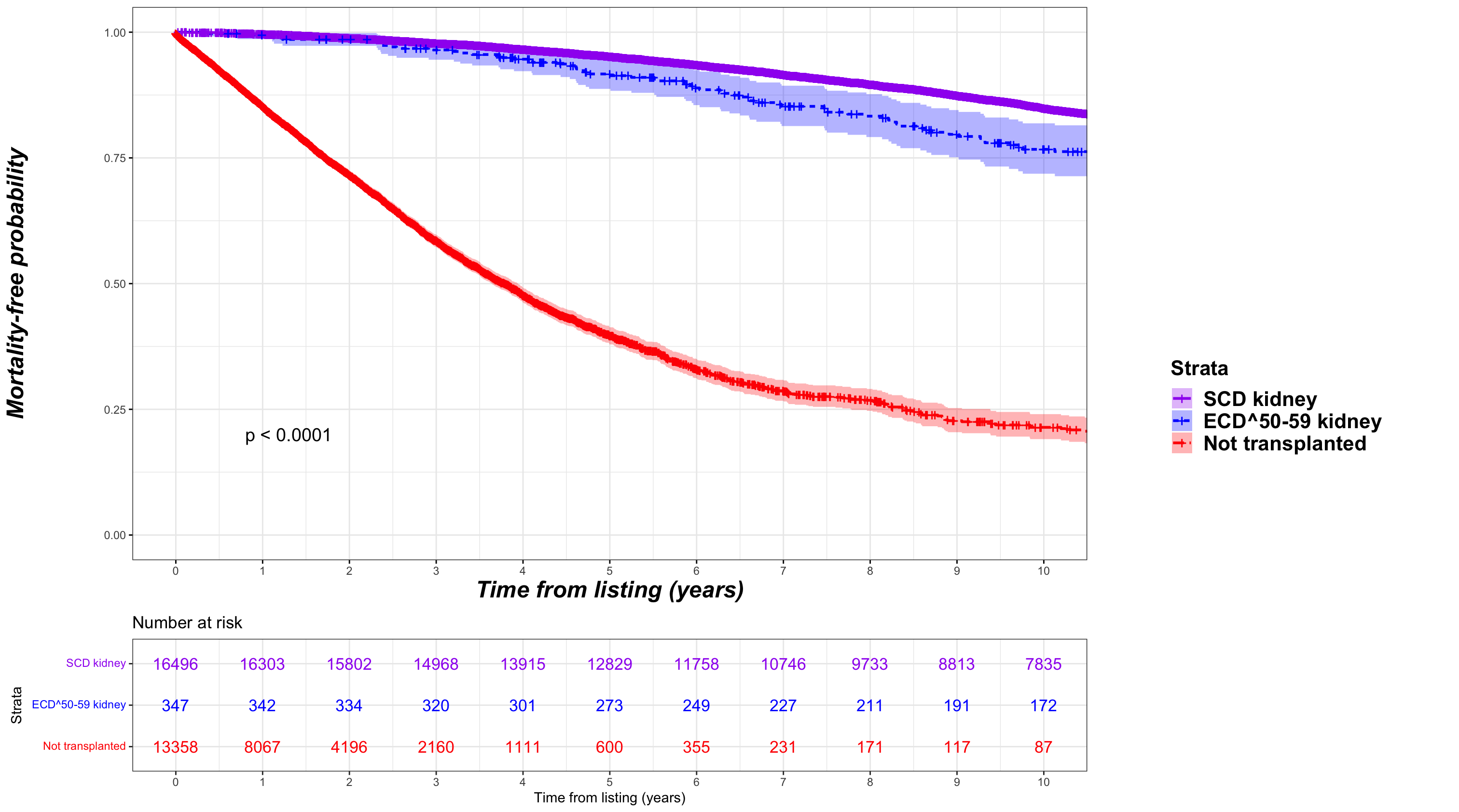

Supplement: Supplementary file 1 [file Image1.JPEG]
